# *Salmonella* effector SopF regulates PANoptosis of intestinal epithelial cells to aggravate systemic infection

**DOI:** 10.1080/19490976.2023.2180315

**Published:** 2023-02-20

**Authors:** Haibo Yuan, Liting Zhou, Yilin Chen, Jiayi You, Hongye Hu, Yuanyuan Li, Rui Huang, Shuyan Wu

**Affiliations:** a Department of Medical Microbiology, School of Biology and Basic Medical Science, Suzhou Medical College of Soochow University, Suzhou, China; b Department of Medical Technology, Suzhou Vocational Health College, Suzhou, China; c Suzhou Key Laboratory of Pathogen Bioscience and Anti-infective Medicine

**Keywords:** *Salmonella* effector SopF, intestinal epithelial cells, PANoptosis, Caspase-8, PDK1-RSK

## Abstract

SopF, a newly discovered effector secreted by *Salmonella* pathogenicity island-1 type III secretion system (T3SS1), was reported to target phosphoinositide on host cell membrane and aggravate systemic infection, while its functional relevance and underlying mechanisms have yet to be elucidated. PANoptosis (pyroptosis, apoptosis, and necroptosis) of intestinal epithelial cells (IECs) has been characterized as a pivotal host defense to limit the dissemination of foodborne pathogens, whereas the effect of SopF on IECs PANoptosis induced by *Salmonella* is rather limited. Here, we show that SopF can attenuate intestinal inflammation and suppress IECs expulsion to promote bacterial dissemination in mice infected with *Salmonella enterica* serovar Typhimurium (*S*. Typhimurium). We revealed that SopF could activate phosphoinositide-dependent protein kinase-1 (PDK1) to phosphorylate p90 ribosomal S6 kinase (RSK) which down-regulated Caspase-8 activation. Caspase-8 inactivated by SopF resulted in inhibition of pyroptosis and apoptosis, but promotion of necroptosis. The administration of both AR-12 (PDK1 inhibitor) and BI-D1870 (RSK inhibitor) potentially overcame Caspase-8 blockade and subverted PANoptosis challenged by SopF. Collectively, these findings demonstrate that this virulence strategy elicited by SopF aggregates systemic infection via modulating IEC PANoptosis through PDK1-RSK signaling, which throws light on novel functions of bacterial effectors, as well as a mechanism employed by pathogens to counteract host immune defense.

## Introduction

*Salmonella enterica* serovar Typhimurium (*S*. Typhimurium) is one of the most common foodborne pathogenic bacteria causing diarrhea in a broad range of hosts. The emergence of antibiotic-resistant *S*. Typhimurium represents a major public health issue.^1^ On the other hand, *S*. Typhimurium is also a useful model organism for investigating the mechanisms of host–bacterium interactions.^2^ It would be potentially significant to provide new paradigms for interactions between bacteria and host immune response, which would provide novel insights for controlling *S*. Typhimurium infection and other infectious diseases.

As an enteric pathogen, *S*. Typhimurium invades into the host initially through intestinal epithelial cells (IECs). *Salmonella* pathogenicity island-1 type III secretion system (T3SS1) and its effectors counteract potent inflammatory responses, enabling *S*. Typhimurium to enter into IECs.^3^ Intriguingly, SopD exerts both pro- and anti-inflammatory response by targeting the Rab8 GTPase.^4^ SopB, SopE, and SopE2 elicit membrane ruffling and fuel cooperative invasion through ADP-ribosylation factor 1 (Arf1) and ras-related C3 botulinum toxin substrate 1 (Rac1) dependent actin polymerization.^5^ In contrast, SipA and SipC can directly target actin cytoskeleton to drive cell invasion.^6,7^ Recent reports demonstrated an additional role of SopE in autophagy flux inside IECs. SopE modulates autophagy flux by interacting with autophagy regulator SP1 when *Salmonella* is located within *Salmonella*-containing vacuole (SCV).^8^ SopF, a newly discovered T3SS1 effector, is not involved in bacterial invasion into epitheliums but targets host cell membranes via phosphoinositide (PIP) interactions to maintain the integrity of nascent SCV.^9^ Moreover, SopF was required for its replication within HeLa cells and full virulence in mice infected with *S*. Typhimurium.^10,11^ How does SopF, a PIP binding effector, make it easy for bacterial dissemination?

IECs *per se* not only serve as mechanical barriers to protect the mucosa from pathogen invasion but also coordinate a number of innate immune defenses.^12^ One possible mechanism linking IECs to bacterial infection is the process of inflammatory programmed cell death (PCD) pathways. PANoptosis is defined as an inflammatory PCD with key features of pyroptosis, apoptosis, and/or necroptosis. PANoptosis of IECs is known to be critically involved in host defense against *S*. Typhimurium infection,^13–16^ which is often referred to as a double-edged sword from the standpoint of pathogen and host survival. Pyroptosis and apoptosis drive the expulsion of infected IECs to limit *S*. Typhimurium replication.^16,17^ The dead and extruded IECs elicit shrinkage of the neighboring epitheliums to maintain intestinal barrier integrity.^18^ In contrast, necroptosis of IECs contributes to intestinal barrier disruption and facilitates *S*. Typhimurium spread to lamina propria.^19^ It remains to be clarified how *S*. Typhimurium conquers PANoptosis of IECs for survival.

Caspase-8 was originally identified as an extrinsic initiator Caspase of apoptosis. Active Caspase-8 transmits the death signals by cleaving Caspase-3 and Caspase-7, which subsequently stimulate various intracellular molecules to induce apoptotic cell death.^20^ Despite its role in promoting apoptosis, Caspase-8 has also been implicated in the onsets of necroptosis and pyroptosis.^21–23^ Research over the last decade has clearly demonstrated that Caspase-8 had a rather pro-survival function by suppressing necroptosis.^22^ Caspase-8 was able to cleave receptor-interacting protein kinase 1 (RIPK1) and RIPK3 and thus prevented the initiation of necroptosis.^24^ Moreover, Gasdermin D (GSDMD), the executor of pyroptosis, could be directly cleaved at aspartate 88 (D88) by Caspase-8, which was important in host defense against bacterial infection.^21^ Additionally, evidence showed that autophagosomal membranes can serve as a platform for Caspase-8 activation.^25^ Overall, Caspase-8 is tightly regulated for its key role in cell death. Interestingly, an intrinsic mechanism, phosphoinositide-dependent protein kinase-1 (PDK1)-p90 ribosomal S6 kinase (RSK) pathway, was revealed to subvert Caspase-8 blockade of necroptosis.^26^ Based on the phosphoinositide targeting function of SopF, we hypothesized that SopF might activate the host PDK1-RSK signaling pathway, and thus affects Caspase-8 function.

Herein, by using a streptomycin-pretreated murine model, we revealed that SopF was required for *S*. Typhimurium to evade host epithelial defense. Mechanistically, we uncovered that SopF activated PDK1-RSK signaling to inhibit Caspase-8 activation in IECs, which resulted in IECs PANoptosis (halted pyroptosis and apoptosis, but promoted necroptosis) and aggravation of systemic infection.

## Results

### S. Typhimurium effector SopF inhibits intestinal inflammation to aggravate systemic infection

**Figure 1.**
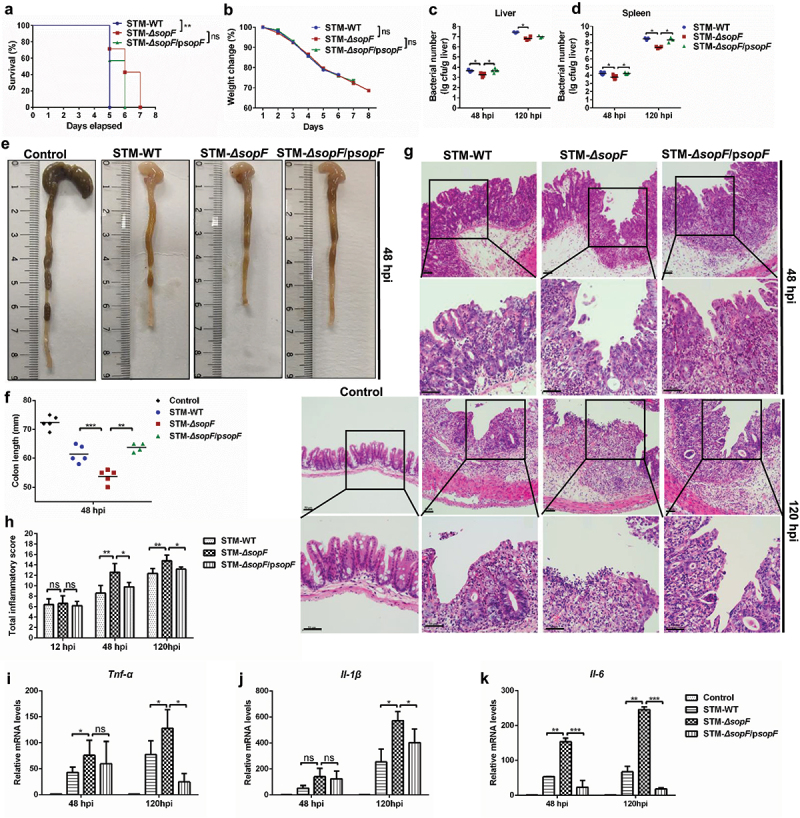
SopF inhibits intestinal inflammation to aggravate systemic infection. Streptomycin-pretreated C57BL/6 mice were orally infected with 5 × 10^7^ CFU of STM-WT, STM-*ΔsopF* or STM-*ΔsopF*/p*sopF*. (a) Survival curve, n = 7 mice/group. (b) Body weight change, n = 10 mice/group. (c, d) The bacterial loads of livers (i) and spleens (j) at 48 hpi and 120 hpi, each data point represents one mouse. (e) Macroscopic pictures of the intestinal tract at 48 hpi, n = 5 mice/group. (f) Quantification of colon length above. (g) Histopathological analysis of the ceca at 48 hpi and 120 hpi (scale bar: 50 μm), n = 5 mice/group. (h) Total inflammatory score of ceca. (i-k) Gene transcription analysis of *Il-6, Tnf-α* and *Il-1β* at 48 hpi and 120 hpi, n = 5 mice/group. Gene expression levels are shown relative to *Gapdh*. Data were compared by one-way ANOVA. Values are expressed as the means ± SD, and statistically significant differences are indicated. *P < .05, **P < .01, ***P < .001, ns: not significant.

To investigate the potential role of SopF in pathogenesis of *S*. Typhimurium, we first monitored mortality and body weight change during the course of infection. Streptomycin pretreatment mice were orally infected with STM-WT, STM-*ΔsopF*, or STM-*ΔsopF*/p*sopF*.^27^ The individuals had a variety moribund state at 4 dpi, and mice gavaged with STM-WT succumbed to infection on the next day. During the same time 70% of the STM-*ΔsopF*-infected mice were still alive. In addition, mice infected with STM-*ΔsopF*/p*sopF* succumbed to infection within 6 d (Figure 1(a)). A gradual decrease in body weight in mice was observed as the infection processed, but without showing any significant difference among the three groups (Figure 1(b)). The accelerated mortality of mice highlights the importance of SopF to pathogenesis of *S*. Typhimurium in early innate immune responses, which in turn greatly affects the outcome of the infection. Consistent with previous research,^10,11^ mice infected with *S*. Typhimurium carrying *sopF* had about two fold of bacterial burden in their livers and spleens at 48 hpi compared with those infected with STM-*ΔsopF*, the marked increase in the bacterial burden is observed at 120 hpi (Figure 1(c-d)). These data confirm that SopF is required for *S*. Typhimurium dissemination in extra-intestinal organs.

Intestinal inflammatory response in intestinal tract is important for host–pathogen interaction.^4^ We next focus on the effects of SopF on *S*. Typhimurium-induced intestinal inflammation. Importantly, from 12 hpi, we observed that the cecal content of mice infected with STM-*ΔsopF* were greatly lost, and the ceca became almost transparent and full of bubbles compared to those infected with *S*. Typhimurium carrying *sopF* (Supplementary Figure 1(a)). The contraction of the colon length observed at 48 hpi in mice infected with STM-*ΔsopF* is greater than those in STM-WT and STM-*ΔsopF*/p*sopF* infected mice (Figure 1(e-f)). We next performed Mayer’s hematoxylin and eosin (H&E) analysis, no obvious lesions were observed at 12 hpi for all strains (Supplementary Figure 1(b)). We found progressive pathological lesions including swelling and infiltration of inflammatory cells into the lamina propria and submucosa in mice infected with all *S*. Typhimurium strains. Importantly, the SopF deficiency exacerbated *S*. Typhimurium-induced histopathological injury of the ceca, including IEC expulsion at 48 hpi and loss of IECs from the mucosal tissue at 120 hpi (Figure 1(g-h)).^28^ To further characterize the intestinal inflammatory responses presented by SopF, we analyzed the level of inflammatory cytokines that are intimately relevant to host defense against intracellular pathogens.^4^ As shown in Figure 1(i-k), the transcription levels of *Il-6, Tnf-α* and *Il-1β* were most prominently induced in cecum with the lastingness of infection, and significantly increased expression of cytokines was observed in the ceca of mice infected with STM-*ΔsopF* compared with those in STM-WT and STM-*ΔsopF* /p*sopF* infected mice at 120 hpi. Collectively, these results suggest that SopF inhibits intestinal inflammation to aggravate systemic infection in response to *S*. Typhimurium infection.

### S. Typhimurium effector SopF restricts the dislodging of IECs to promote bacterial dissemination

**Figure 2.**
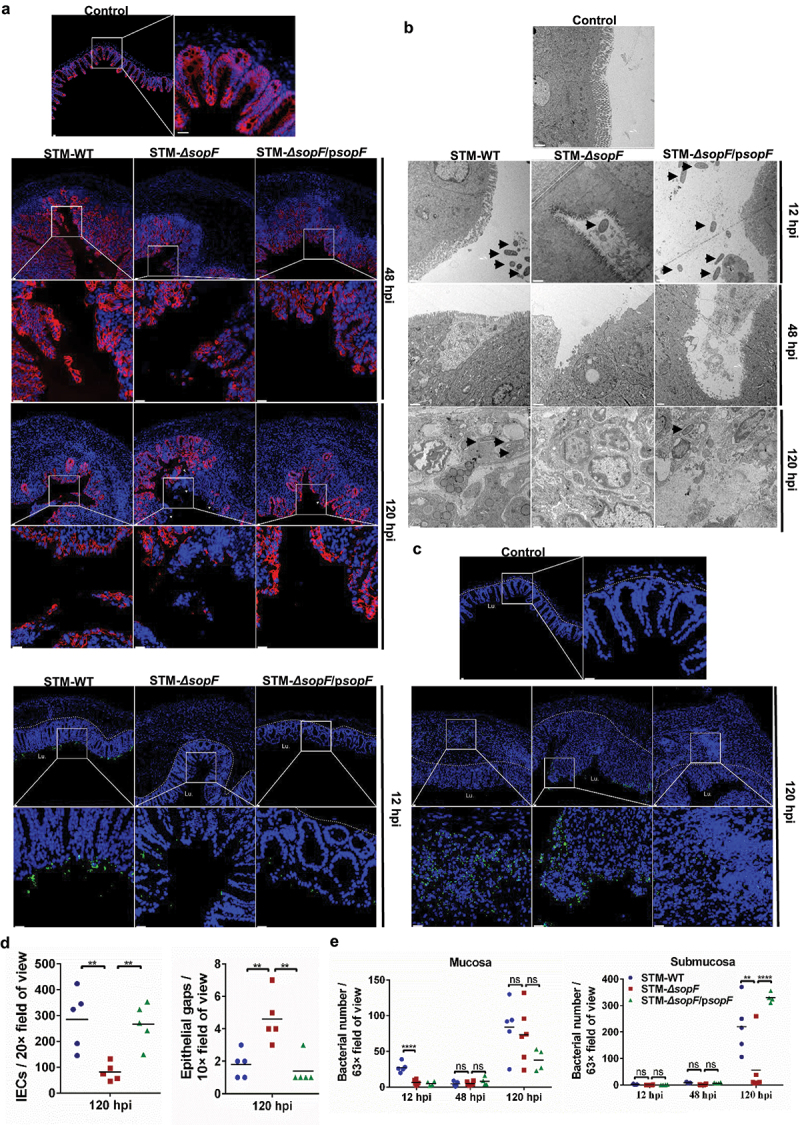
SopF restricts the dislodging of IECs to promote bacterial dissemination. Streptomycin-pretreated C57BL/6 mice were orally infected with 5 × 10^7^ CFU of STM-WT, STM-*ΔsopF* or STM-*ΔsopF*/p*sopF*. n = 5 mice/group. (a) Immunofluorescence analysis of EpCAM (red) at 48 hpi and 120 hpi. Nuclei were counterstained with DAPI (blue). White arrowheads indicate epithelial gaps. (Scale bar: 20 μm). (b) Representative images of transmission electron microscopy (TEM) analysis of the ceca at 12 hpi, 48 hpi and 120 hpi (Scale bar: 1 μm). Arrows indicated the bacteria. (c) Distribution of *S*. Typhimurium (green) in ceca at 12 hpi and 120 hpi. Nuclei were counterstained with DAPI (blue) (Scale bar: 20 μm). Dashed line indicates the end of mucosa. (d) Microscopy-based quantification of epithelial gaps per 10× field of view and IEC numbers per 20× field of view. (e) Quantification of bacterial number in mucosa and submucosa.

IECs negotiate essential roles in separating the microbial community of the lumen from the sterile systemic milieu. Expulsion of infected IECs restricts the pathogen’s intraepithelial proliferation, and serves as a general defense mechanism against enteric pathogen infection.^16,29^ To determine the interaction between *S*. Typhimurium and IEC mediated by SopF, we employed fluorescence microscopy to visualize the integrity of the IEC barrier and the distribution of *S*. Typhimurium. In accordance with the histopathological examination, staining for the epithelial marker EpCAM was clear and intact at 12 hpi (Supplementary Figure 1(c)). Accordingly, we observed significant loss of total IEC numbers and more epithelial gaps per 10x field of view in mucosal tissue of mice infected with STM-*ΔsopF* at 120 hpi (Figure 2(a-d)). We further visualized the ultrastructure of ceca with transmission electron microscope (TEM) (Figure 2(b)). Bacteria were seen in the intestinal lumen at the early stage of infection (12 hpi) (Figure 2(b-c)). To assess the possible correlation between IEC expulsion and the onset of bacterial dissemination, we extend these studies by monitoring the time course of the infection. At 48 hpi, anomalies of the brush border structures, such as disordered, fewer, and shorter microvilli, were observed in mice ceca infected with STM-*ΔsopF*. In contrast, mice infected with *S*. Typhimurium carrying *sopF* appeared minor injury in IECs at 48 hpi but significantly higher bacteria load in the submucosa at 120 hpi (Figure 2(b)). Anti-*S*. Typhimurium-LPS staining revealed an increase in colonization of bacteria at 12 hpi (Figure 2(c)), and a minor increase at 48 hpi (Supplementary Figure 1(d)) in ceca of mice infected with STM-*ΔsopF*. At the late stage of infection (120 hpi), *S*. Typhimurium carrying *sopF* predominantly colonized the submucosa, while STM-*ΔsopF* was still in the mucosa (Figure 2(c-e)). Together, these results suggest that SopF restricts IEC expulsion to promote bacterial dissemination.

### S. Typhimurium effector SopF regulates the PANoptosis of intestinal epithelial cells in host defense against S. Typhimurium infection

**Figure 3.**
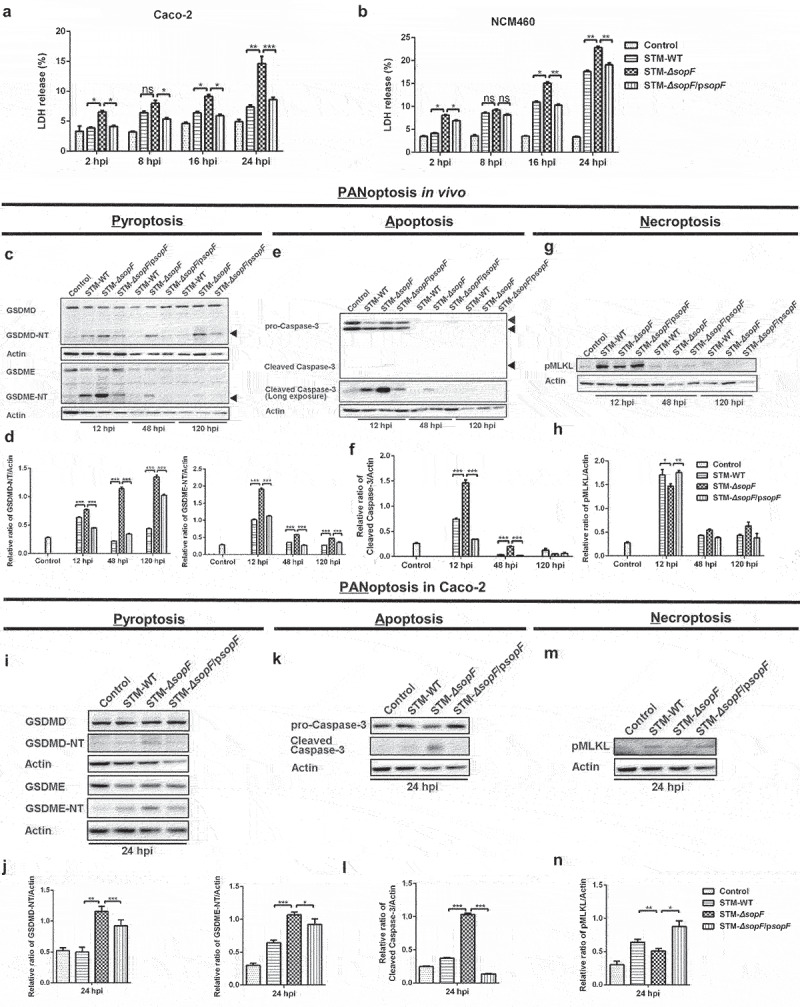
SopF regulates the PANoptosis of intestinal epithelial cells in host defense against *S*. Typhimurium infection. (a, b) Caco-2 cells (a) or NCM460 (b) cells were infected with STM-WT, STM-*ΔsopF* or STM-*ΔsopF*/p*sopF* at an MOI of 100. LDH release were detected at 2 hpi, 8 hpi, 16 hpi and 24 hpi. (c-h) Streptomycin-pretreated C57BL/6 mice were orally infected with 5 × 10^7^ CFU of STM-WT, STM-*ΔsopF* or STM-*ΔsopF*/p*sopF*. IECs were isolated from ceca of mice at 12 hpi, 48 hpi and 120 hpi. Western blot analysis of isolated IEC lysates with specific antibodies to (c) GSDMD/GSDMD-NT, GSDME, GSDME-NT; (e) pro-Caspase-3/cleaved Caspase-3; (g) pMLKL and the control Actin. (d, f, h) Quantification of western blot analysis above. (i-h) Caco-2 cells were infected with STM-WT, STM-*ΔsopF* or STM-*ΔsopF*/p*sopF* at an MOI of 100 at 24 hpi. Western blot analysis of whole cell lysates with specific antibodies to (i) GSDMD/GSDMD-NT, GSDME, GSDME-NT; (k) pro-Caspase-3/cleaved Caspase-3; (m) pMLKL and the control Actin. (j, l, n) Quantification of western blot analysis above. Data were compared by one-way ANOVA. Values are expressed as the means ± SD, and statistically significant differences are indicated. ***P < .001; **P < .01; *P < .05. Data were from at least three experiments.

Although epithelium is primarily considered as a mechanical barrier, studies have indicated that the cell fate of IECs allows them to counteract pathogens.^19,30^ We investigated the role of SopF in cell death of IECs in response to *S*. Typhimurium infection. A reduced release of lactate dehydrogenase (LDH) was found in the human colon carcinoma Caco-2 cells and human normal colonic epithelial NCM460 cells infected with *S*. Typhimurium carrying *sopF*, revealing that SopF suppressed the cell death of IECs (Figure 3(a-b)). Recent evidence has shown an interplay between pathogens and hosts with respect to PANoptosis.^31^ To assess the effects of SopF on PANoptosis of IECs, we isolated IECs from the ceca of *S*. Typhimurium-infected mice. Western blot analysis showed a higher protein level of GSDMD-NT fragments, which can form membrane pores to induce pyroptosis, in STM-*ΔsopF*-infected mice. Epithelial-derived GSDME that is cleaved by Caspase-3 also participates in the pathogenesis of intestinal inflammation.^32^ Accordingly, a robust GSDME activation was found in STM-*ΔsopF*-infected mice (Figure 3(c-d)). The aforementioned results suggest that SopF inhibits pyroptosis of IECs. In addition to pyroptosis, we find an increased cleavage of apoptotic executor caspase, Caspase-3, in mice infected with STM-*ΔsopF* (Figure 3(e-f)). Pyroptotic and apoptotic IECs apicaled-out to maintain epithelial barrier integrity, while SopF prevented the elimination of infected IECs.^15,33^ Necroptosis of IECs also leads to epithelial barrier dysfunction that promotes *S*. Typhimurium dissemination.^19^ Next, we examined whether SopF was also involved in the necroptosis of IECs. As expected, robust phosphorylation of mixed lineage kinase domain-like protein (MLKL) was observed in mice infected with *S*. Typhimurium carrying *sopF* at 12 hpi, suggesting that SopF promotes necroptosis of IECs, which enables the dissemination of *S*. Typhimurium to lamina propria or even extra-intestinal organs (Figure 3(g-h)). Similar results were shown in Caco-2 cells (Figure 3(i-n)) and NCM460 cells (Supplementary Figure 2(a-f)). Collectively, these data indicate that SopF modulates PANoptosis by inhibiting apoptosis and pyroptosis but facilitating the necroptosis of IECs, which may relate to the *S*. Typhimurium systemic infection.

### S. Typhimurium effector SopF inhibits the activation of Caspase-8, a molecular switch for PANoptosis

**Figure 4.**
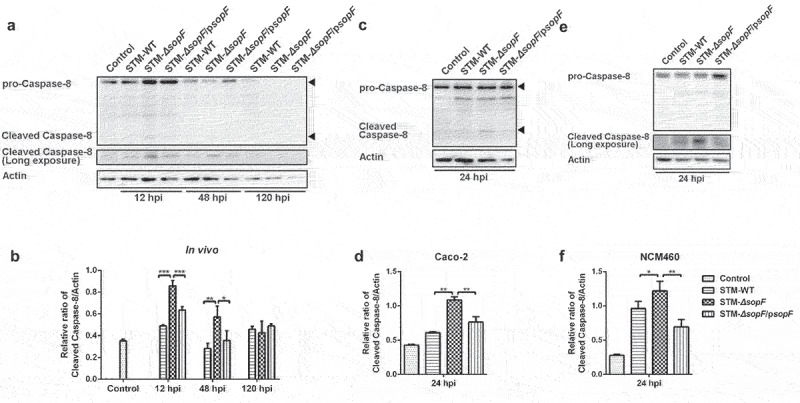
SopF inhibits the activation of Caspase-8, a molecular switch for PANoptosis. (a, b) Streptomycin-pretreated C57BL/6 mice were orally infected with 5 × 10^7^ CFU of STM-WT, STM-*ΔsopF* or STM-*ΔsopF*/p*sopF*. IECs were isolated from ceca of mice at 12 hpi, 48 hpi and 120 hpi. (c, d) Caco-2 cells (c) or NCM460 cells (e) were infected with STM-WT, STM-*ΔsopF* or STM-*ΔsopF*/p*sopF* at an MOI of 100 at 24 hpi. Western blot analysis of isolated IEC lysates (a) or whole cell lysates (c, e) with specific antibodies to pro-Caspase-8 and cleaved Caspase-8. (b, d, f) Quantification of cleaved Caspase-8. Data were compared by one-way ANOVA. Values are expressed as the means ± SD, and statistically significant differences are indicated. ***P < .001; **P < .01; *P < .05. Data were from at least three experiments.

Caspase-8, activated by cell surface death receptor ligation and oligomerization, is a molecular switch for apoptosis, necroptosis, and pyroptosis.^34^ Besides, Caspase-8 orchestrate epithelial PCD and prevents barrier dysfunction in response to *S*. Typhimurium infection.^19^ To understand the potential impact of SopF on Caspase-8, we tested the isolated IECs from the ceca of *S*. Typhimurium-infected mice. Increased cleavage of Caspase-8 was observed in mice infected with *Salmonella* lacking *sopF* compared with those infected with WT or complemented mutant strains at 12 and 48 hpi. (Figure 4(a-b)). Correspondingly, the deletion of *sopF* resulted in an increased cleavage of Caspase-8 at 24 hpi in Caco-2 cells and NCM460 cells (Figure 4(c-f)). Overall, these data indicate that SopF abrogates the activation of Caspase-8 to modulate IECs PANoptosis.

### PDK1-RSK signal is required for Caspase-8 blockade during SopF-mediated S. Typhimurium infection

**Figure 5.**
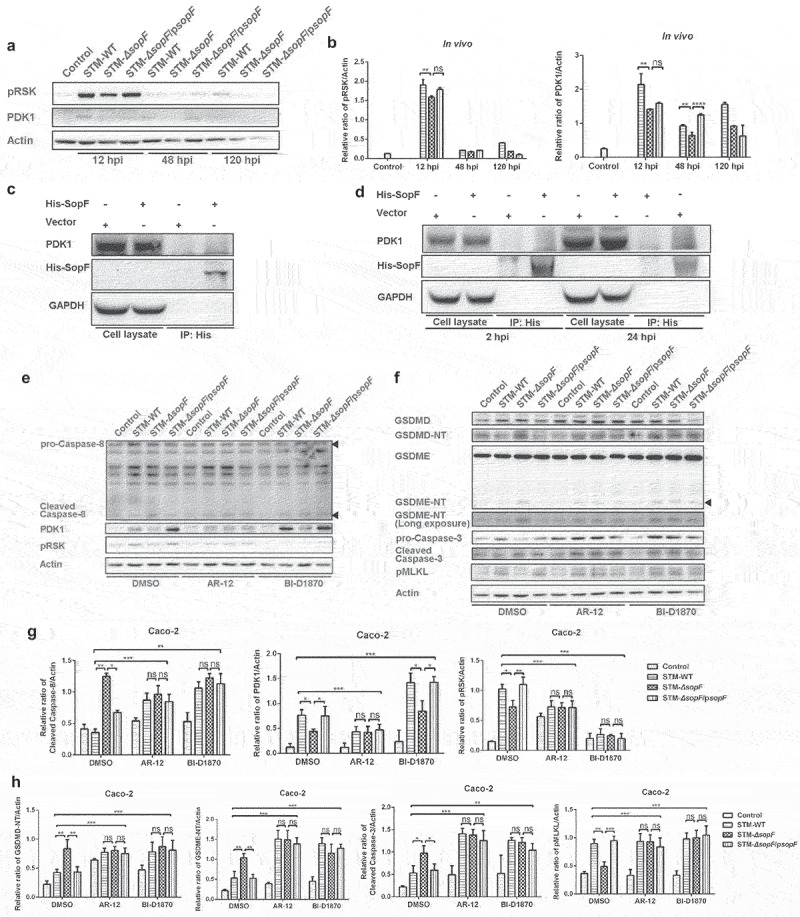
PDK1-RSK signal is required for Caspase-8 blockade during SopF-mediated *S*. Typhimurium infection. (a, b) Streptomycin-pretreated C57BL/6 mice were orally infected with 5 × 10^7^ CFU of STM-WT, STM-*ΔsopF* or STM-*ΔsopF*/p*sopF*. IECs were isolated from ceca of mice at 12 hpi, 48 hpi and 120 hpi. (c) 293 T cells were transfected with an empty vector or pcDNA3.1-SopF-6*His-T2A-GFP for 48 h. (d) Caco-2 cells were infected with STM-*ΔsopF* carrying an empty vector or a vector expressing SopF: His at an MOI of 100 for 2 hpi and 24 hpi. (c and d) His-tagged SopF were immunoprecipitated from cell lysates and assessed for their ability to bind endogenous PDK1. (e-h) After being pre-treated with mock (DMSO, 1 μL/mL), PDK1 inhibitor (AR-12, 1 μM) or RSK inhibitor (BI-D1870, 10 μM) for 1 h, Caco-2 cells were infected with STM-WT, STM-*ΔsopF* or STM-*ΔsopF*/p*sopF* at 24 hpi. Western blot analysis of whole cell lysates with specific antibodies to (e) pro-Caspase-8, cleaved Caspase-8, PDK1 and pRSK; (f) GSDMD/GSDMD-NT, GSDME, GSDME-NT, pro-Caspase-3/cleaved Caspase-3; pMLKL and the control Actin. (g, h) Quantification of western blot analysis above. Data were compared by one-way ANOVA. Values are expressed as the means ± SD, and statistically significant differences are indicated. ***P < .001; **P < .01; *P < .05; ns: not significant. Data were from at least three experiments.

Caspase-8 may function as a gatekeeper to regulate PANoptosis. Previous work by Yang et al. has shown that the 3-phosphoinositide-dependent protein kinase 1 (PDK1)-ribosomal S6 kinase (RSK) signal is an intrinsic mechanism for diminishing the Caspase-8 blockade.^26^ Of note, SopF binds to the phosphoinositide of the host cell membrane to facilitate the stability of nascent *Salmonella*-containing vacuole (SCV).^9^ Thus, we hypothesized that PDK1-RSK signaling was involved in the SopF-mediated blockade of Caspase-8 activation. Western blot analysis revealed that the protein levels of PDK1 and phosphorylated RSK were potentially higher in mice infected with *S*. Typhimurium carrying *sopF* than those in STM-*ΔsopF*-infected groups (Figure 5(a-b)), as well as in Caco-2 cells (Supplementary Figure 3(a, b)) and NCM460 cells (Supplementary Figure 3(c, d)), suggesting that SopF could activate PDK1-RSK pathway to abolish Caspase-8 activation. His or His-SopF, expressed in 293 T cells, was immunoprecipitated and assessed for its ability to interact with endogenous PDK1. Results showed that PDK1 was undetected in His-SopF immunoprecipitated samples (Figure 5(c)). We then tested whether SopF:His, translocated by *S*. Typhimurium, could interact with endogenous PDK1 in infected Caco-2 cells. STM-*ΔsopF* carrying empty pBAD (STM-*ΔsopF*/pBAD) or pBAD for His-tagged SopF expression (STM-*ΔsopF*/p*sopF*:His) were constructed to establish the infection model. The PDK1 was also undetected in SopF:His immunoprecipitated samples both at early and late stages of infection, suggesting that SopF could not directly interact with PDK1 (Figure 5(d)).

AR-12 and BI-D1870 are ATP antagonists for PDK1 and RSK, respectively.^35,36^ The PDK1 inhibitor AR-12 potentially eliminated the increased phosphorylation of RSK but subverted the decreased activation of Caspase-8 presented by SopF at 24 hpi. Moreover, the RSK inhibitor BI-D1870 had the same effect on Caspase-8 in Caco-2 cells infected with *S*. Typhimurium (Figure 5(e-g)). The results indicate that SopF inhibits the cleavage of Caspase-8 through PDK1-RSK signaling pathway. Given the fact that Caspase-8 played a central role in regulating PANoptosis, we further pretreated Caco-2 cells with either AR-12 or BI-D1870. Western blot analysis showed that the protein levels of GSDMD-NT, GSDME-NT, as well as, cleaved Caspase-3 were increased, and the phosphorylation of MLKL was decreased after administration of AR-12 and BI-D1870. No statistical differences were observed among the three infection groups after the treatment of inhibitors. The data indicate that both AR-12 and BI-D1870 restore the halted pyroptosis and apoptosis and the prompted necroptosis (Figure 5(f-h)).

SopF was reported to decrease LC3 decoration by targeting ATP6V0C in the V-ATPase for ADP-ribosylation at the initial stage of xenophagy.^9^ Autophagosomal membranes have been reported to provide a platform for activation of Caspase-8^25^. To our surprise, the administration of Bafilomycin A1, a V-ATPase inhibitor, was unable to restore Caspase-8 blockade presented by SopF, indicating that LC3-II accumulation was dispensable for SopF-mediated Caspase-8 inactivation (Supplementary Figure 4(a-d)). Thus, our data demonstrated that PDK1-RSK signaling that was activated by SopF was the upstream of Caspase-8.

## Discussion

IECs are the gatekeepers for *S*. Typhimurium invasion. In addition to acting as a mechanical barrier, IECs also participate in host immune defense. Intestinal pathogens like *S*. Typhimurium have evolved strategies to counteract this host defense. SopF is a newly discovered effector secreted by *Salmonella* T3SS1. It was reported that SopF could promote bacterial dissemination in mice.^9^ In accordance with previous work, our work validated that mice were susceptible to *S*. Typhimurium carrying *sopF* infection, as observed by increased mortality and bacterial loads in systemic organs. SopF exhibited an anti-inflammatory role in the intestinal tract, as visualized by shorter ceca, severe pathological lesions, and higher levels of inflammatory cytokines in mice infected with STM-*ΔsopF*. Taken together, these results demonstrated that SopF ameliorated gut inflammation and aggravated system infection.

Notably, *sopF* deletion mutant only partially reduced the bacterial loads in both liver and spleen, indicating that SopF was necessary but not sufficient for bacterial dissemination. Many additional effectors have been demonstrated to contribute significantly to *S*. Typhimurium systemic infection.^37–39^ Our previous work showed that another *S*. Typhimurium virulence determinate, SpvC, attenuated intestinal inflammation to aggravate systemic infection like the effect of SopF in this study. However, the mechanisms were vastly different.^40^ SpvC, mainly secreted by T3SS2, was found to interrupt the anti-bacterial function of macrophages. Considering that T3SS1 effectors are generally associated with bacterial entry into non-phagocytes, we next focus on the lesions of IECs affected by SopF. Previous publications highlighted the role of IECs expulsion in restricting bacterial colonization.^15,16^ However, this process may help *S*. Typhimurium complete its infectious cycle and also contribute to boosted recolonization of the enteroid lumen.^41,42^ These observations may correlate with other noted mechanisms for luminal population restriction, such as commensal microbiota competition.^43^ We observed reduced IEC expulsion but increased bacterial migration into the submucosa via morphological observation in mice ceca infected with *S*. Typhimurium carrying *sopF*. These results indicate that SopF-mediated IEC expulsion plays a critical role during *S*. Typhimurium infection.

Inflammatory programmed cell death in intestinal epithelium cells involves in multiple signaling pathways that are activated during the whole stage of *S*. Typhimurium infection, and the regulation of these signaling pathways has been a useful strategy for *S*. Typhimurium to escape from the host immune system.^44^ It is generally accepted that the PANoptosis (pyroptosis, apoptosis, and necroptosis) of IECs plays a critical role during the course of *S*. Typhimurium infection.^15,19,45–47^ *S*. Typhimurium T3SS1 effectors, SipB and SipD, were reported to activate Caspase-3 by impeding the translocation of NF-κB subunit p65 into the nucleus and thereby induce apoptosis.^48^ The T3SS2 effectors, SseK1 and Ssek3, could directly target the death domain of TRADD and inhibit necroptotic cell death in *Salmonella*-infected macrophages.^49,50^ Our findings demonstrate that SopF inhibits pyroptosis and apoptosis but promotes necroptosis of IECs *in vivo*. This notion was supported by data in epithelial cell lines Caco-2 and NCM460 cells. Collectively, our results indicate that SopF manipulates PANoptosis of IECs.

Caspase-8 not only promotes apoptosis but also inhibits necroptosis by suppressing the function of RIPK to activate MLKL, an executor of necroptosis.^24^ Recent investigations have discovered additional functions of Caspase-8 for the regulation of inflammation in various ways depending on its catalytic activity and scaffolding role.^51^ Taken together, Caspase-8 is defined as the molecular switch for PANoptosis.^24,34^ Our data suggest that SopF inhibits Caspase-8 activation to regulate the PANoptosis of IECs.

To ensure the best bacterial growth inside the cell, *Salmonella* had developed a series of strategies to escape the host immune system. SopB can decrease gut inflammation via activating Akt signaling to inhibit PANoptosis of host cells.^52–54^ Furthermore, Akt activation was blocked by the presence of PDK1 inhibitor, suggesting that PDK1-Akt signaling pathway was responsible for *Salmonella* induced cell death.^53^ Previous studies have shown that SopF can bind PIP to the host cell membrane, which may lead to the activation of PDK1.^9,55^ We found that the protein level of PDK1 was higher in mice infected with STM-WT, as well as increased phosphorylation of RSK, which inhibited Caspase-8 activation.^26^ Immunoprecipitation evidence showed that PDK1 was not an interaction partner of SopF, providing additional evidence that SopF targeted PIP to activate PDK1. Accordingly, SopF-mediated Caspase-8 inactivation was restored after the administration of PDK1 and RSK inhibitors. PDK1 phosphorylates Ser221 in the N-terminal kinase domain (NTKD) of RSK, while extracellular signal regulated kinase (ERK) permits Ser380 site phosphorylation in the C-terminal kinase domain (CTKD).^36^ As our experiments focus on the role of RSK Ser221, we shall not rule out the possibility of an ERK pathway in mediating RSK phosphorylation. In addition to RSK, AKT and protein kinase C (PKC) are also well-established activation molecules downstream of PDK1.^56^ PKC phosphorylated NLRC4 is critical for inflammasome activation in response to *S*. Typhimurium infection.^57^ This may imply that SopF inhibits PDK1 activation, leading to blockade of NLRC4 inflammasome and thus inhibiting pyroptosis of IECs.

Xenophagy, a member of noncanonical autophagy, could serve as host defense to capture intracellular pathogens and subject them to lysosomal clearance.^10^ Bafilomycin A1 inhibits V-ATPase acidification independently of its effect on autolysosome fusion, implying that this drug could disrupt both canonical autophagy and xenophagy.^58^ In canonical autophagy, the inhibition of autolysosome fusion results in LC3-II accumulation, whereas, the disruption of V-ATPase in xenophagy leads to the inactivation of LC3. LC3-II accumulation in Bafilomycin A1-treated groups indicates that other *S*. Typhimurium effectors could induce canonical autophagy, which, to our knowledge, might mask the contribution of SopF-blocked-xenophagy on Caspase-8.^59^ Therefore, a site-directed mutant strain STM-*ΔsopF*/p*sopF* Y224D, which abolishes its xenophagy-inhibition activity, is needed to rule out its polar effect on other effectors. On the other hand, studies have revealed that autophagy plays a multiple role in Caspase-8 activation, the interaction between Caspase-8 and autophagy needs further study.^60,61^

**Figure 6.**
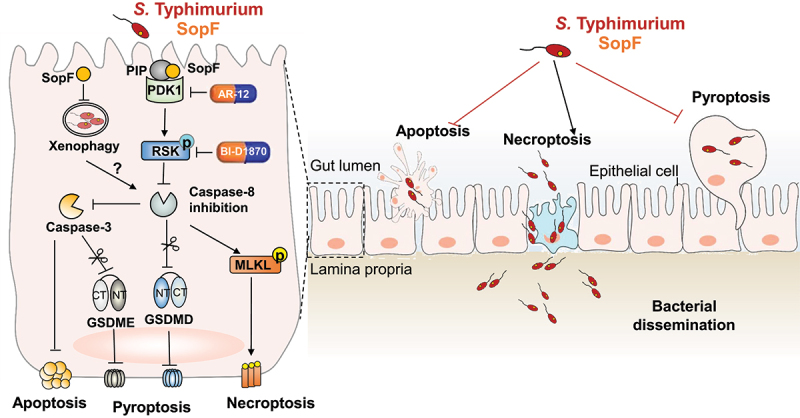
Schematic diagram of SopF regulated PANoptosis during *S*. Typhimurium infection. SopF, a PIP binding effector, inactivates Caspase-8 through PDK1-RSK signaling. The blockade of Caspase-8 down-regulates the cleavage of GSDMD, GSDME and Caspase-3, but up-regulates the phosphorylation of MLKL. These signaling cascade explain the mechanism by which diminished pyroptosis and apoptosis, but facilitated necroptosis. PDK1 inhibitor (AR-12) and RSK inhibitor (BI-D1870) overcome Caspase-8 blockade and restore the IECs PANoptosis. The SopF-halted pyroptosis and apoptosis prevents the expulsion of infected IECs, whereas the SopF-induced necroptosis enables *S*. Typhimurium *per se* spread into lamina propria. This event presented by SopF ameliorated intestinal inflammatory to aggravate systemic infection. PIP, phosphoinositide; PDK1, phosphoinositide-dependent protein kinase-1; RSK, phosphorylate p90 ribosomal S6 kinase; GSDMD, gasdermin D; GSDME, gasdermin E; MLKL, mixed lineage kinase domain-like; IECs, intestinal epithelial cells.

In summary, we identified a novel function of SopF in regulating IECs PANoptosis to aggravate dissemination of *S*. Typhimurium, as a model in Figure 6. In this paradigm, SopF, a PIP binding effector, blocked Caspase-8 activation through PDK1-RSK signaling. Owing to its importance in PCD, Caspase-8 sites as a crossroad to regulate PANoptosis. The suppression of Caspase-8 decreased pyroptosis and apoptosis but increased necroptosis. Blocking potential therapeutic targets of either PDK1 or RSK subverted Caspase-8 blockade and the subsequent IECs PANoptosis. SopF-inhibited pyroptosis and apoptosis impeded the elimination of infected IECs, and SopF-provoked necroptosis potentiated *S*. Typhimurium to internalization thus contributing to bacterial dissemination. These findings reveal a novel mechanism whereby SopF manipulates cell fate decisions of IECs, which may provide attractive therapeutic strategies for the control of *S*. Typhimurium infection and other corresponding diseases.

## Materials and methods

### Bacterial strains and culture conditions

*S*. Typhimurium SL1344 strains harboring the pET28a vector including wild-type (STM-WT), *sopF* deletion mutant (STM-*ΔsopF*), and *sopF* complemented strain (STM-*ΔsopF*/p*sopF*) were kindly supplied by Professor Feng Shao (National Institute of Biological Sciences, Beijing, China).^10^ STM-*ΔsopF* carrying empty pBAD (STM-*ΔsopF*/pBAD) or pBAD for His-tagged SopF expression (STM-*ΔsopF*/p*sopF*:His) were constructed in this study. The primers used for bacterial strain construction are listed in Table 1. Single colonies were inoculated in Luria Bertani (LB, Hangwei, China) broth with shaking at 220 rpm at 37°C. STM-WT, STM-*ΔsopF*, and STM-*ΔsopF*/p*sopF* were cultured in the media with 50 µg/ml of kanamycin (Yeasen, China). STM-*ΔsopF*/pBAD, and STM-*ΔsopF*/p*sopF*: His were cultured in the media containing 100 µg/ml of Ampicillin (Yeasen, China). On the day of infection, STM-WT, STM-*ΔsopF*, STM-*ΔsopF*/p*sopF*, STM-*ΔsopF*/pBAD, and STM-*ΔsopF*/p*sopF*:His were subcultured 1:100 at 37°C in fresh LB broth to log phase. Both STM-*ΔsopF*/pBAD, and STM-*ΔsopF*/p*sopF*:His were supplemented with 0.2% L-arabinose (Sigma, USA). Bacteria were then washed three times in phosphate-buffered saline (PBS), quantified by OD_600_.

**Table 1.** Primers used for construction and identification of strains.

Primers		Sequences*^a^*
*sopF* (*Xho* I)	sense	5’- CCTCGAGatgctcaaacctatctgccat −3’
*sopF* (*Eco*R I)	antisense	5’- GGAATTCatataatattatgcagtctctattaagcgc −3’
*sopF*-ko-verify	sense	5’- CGAAGCCGCAGACCTGATTG −3’
	antisense	5’- CATCGCGGATATTTCCCTTGATC −3’

^a^Underline indicates restriction site.

### Cell culture

NCM460 cells were purchased from the MINGJING BIOLOGY Co., Ltd, and cultured in RPMI 1640 (HyClone Laboratories) supplemented with 10% (v/v) fetal bovine serum (FBS; Biological Industries, Kibbutz Beit-Haemek, Israel). Caco-2 cells were kindly provided by Professor Weiqi He (Soochow University, Suzhou, China) and cultured in complete Dulbecco’s modified Eagle’s medium (DMEM) (HyClone, Logan, Utah, USA) supplemented with 10% (v/v) FBS. All these cells were routinely grown at 37°C in a humidified atmosphere of 5% CO_2_.

### S. typhimurium infection in vivo and ethics statement

Female C57BL/6 mice (6–8 weeks) were bred and maintained at the experimental animal center of Soochow University. All animal experiments were approved by the Ethics Committee of Soochow University and conducted in accordance with the Guidelines for the Care and Use of Research Animals established by Soochow University. The streptomycin-pretreated mouse model was established as described previously.^40^ Mice that fasted for 4 h were administered intragastrically with streptomycin (100 μl of 200 mg/ml solution in sterile water; Sigma, USA). After 24 h, mice were randomly divided into four groups, including control, STM-WT, STM-*ΔsopF*, and STM-*ΔsopF*/p*sopF* infection groups. 100 µL of either 5 × 10^7^ colony-forming unit (CFU) *S*. Typhimurium strains or sterile PBS were used for oral gavage to mice that were fasted for 4 h before infection. Mice were sacrificed using CO_2_ asphyxiation at indicated time points.

For histopathology, tissue samples were harvested and fixed in 4% paraformaldehyde, processed according to standard procedures for dehydration, paraffin embedding, section cutting, and deparaffinization. The sections were stained with hematoxylin–eosin (Baso, Zhuhai, China) and observed under a light microscope (Olympus, Japan). Total inflammatory scores were assessed based on the following parameters according to previous literature:^28^ neutrophil infiltration (0, none; 1, slight increase; 2, marked increase), fibrin deposition, submucosal neutrophil margination, submucosal edema, epithelial necrosis, epithelial ulceration (0, absent; 1, present). The percentage of pathological lesions was counted on a total scale of 0–20.

For bacterial burden measurement, spleen, and liver were harvested and then immersed in 100 µg/ml of amikacin for 1 h. Tissues were homogenized in PBS containing 0.3% Triton (Sigma, USA) for 30 min. CFU values were quantified by plating lysates with appropriate dilutions onto *Salmonella-Shigella* agar (Hangwei, China), followed by incubation overnight.

For immunofluorescence analysis, ceca samples were fixed with 4% paraformaldehyde at 4°C overnight, processed for paraffin embedding. Tissues were cut into 6-μm transverse sections, followed by deparaffinizing and rehydrating for antigen retrieval. The sections were blocked with 3% BSA for 30 min and then incubated with diluted primary antibodies at 4°C overnight. The antibody against EpCAM (#GB11274) was purchased from Servicebio (Wuhan, China; 1:3000 dilution). The antibody against *S*. Typhimurium O antigen (#S10820100) was purchased from the Lanzhou Institute of Biological Products Co., Ltd. (Lanzhou, China; 1:200 dilution). Next, the slides were incubated with Cy3 (#GB21303; 1:300 dilution) or Alexa Fluor 488 (#GB25303; 1:400 dilution) conjugated goat anti-rabbit immunofluorescent secondary antibodies purchased from Servicebio (Wuhan, China). The nuclei were stained with DAPI (#G1012) purchased from Servicebio (Wuhan, China). Slides were mounted in Anti-fade mounting medium (#G1401; Servicebio). Images were photographed using a Nikon microscope (ECLIPSE, Ts2R-FL, Tokyo, Japan).

Sample processing for transmission electron microscopy (TEM) was carried out in the School of Biology and Basic Medical Science, Medical College of Soochow University. Ceca samples were fixed in ice-cold 2.5% glutaraldehyde for at least 4 h. Samples were washed twice using 0.1 M phosphate buffer for 15 min at room temperature. Subsequently, the samples were post-fixed in 1% OsO4 for 1 h, dehydrated through an acetone series and embedded in epoxy resin. Ultra-thin sections were stained and observed using a 120 kV Transmission electron microscope (HT7700, Hitachi, Japan).

### S. Typhimurium infection in vitro

Cells were seeded in 12-well plates (5 × 10^5^ cells/well) and co-cultured with *S*. Typhimurium at a multiplicity of infection (MOI) of 100:1. At 1 hpi, cells were washed with PBS three times and incubated further for 2 h with a fresh medium containing amikacin (100 µg/ml, MilliporeSigma, Burlington, MA, USA) to kill the extracellular bacteria. Afterward, cells were washed again, and a fresh medium containing amikacin (10 µg/ml, MilliporeSigma) was added to limit extracellular replication of bacteria. For inhibition of PDK1 activation, cells were pretreated with AR-12 (1 μM; S1106, Selleck, USA) 1 h before infection. For inhibition of RSK phosphorylation, cells were pretreated with BI-D1870 (10 μM; HY-10510, MCE) 1 h before infection. Cells were pretreated with Bafilomycin A1 (100 nM; ab120497, Abcam) 2 h before infection to interfere with autophagy. Proteins were extracted using a radioimmunoprecipitation assay (RIPA, Beyotime Biotechnology) buffer containing protease inhibitors and phosphatase inhibitors (Beyotime Biotechnology).

### Lactate dehydrogenase (LDH) release assay

Cells were seeded in 12-well plates (4 × 10^5^ cells/well). The infection model was established as described previously. Cell culture supernatant was collected for detection according to manufacturer’s protocol (Beyotime Biotechnology). The absorbance was read at 490 nm with Infinite® F50 Absorbance Microplate Reader (Tecan, Switzerland).

### Transfection experiments

The eukaryotic expression vector pcDNA3.1-SopF-6*His-T2A-GFP was purchased from Tsingke Biotechnology Co., 293 T cells were transiently transfected with plasmids using ExFect® Transfection Reagent (Vazyme, China) for 48 h according to the manufacturer’s instructions.

### Immunoprecipitation

Caco-2 cells were seeded in 10-cm dishes and co-cultured with STM-*ΔsopF*-pBAD or STM-*ΔsopF*/p*sopF*: His at an MOI of 100 for 2 hpi and 24 hpi. Following bacterial infections, cells were washed three times with cold PBS. Protein extracts were incubated with 20 μl protein A/G Plus-Agarose (Santa Cruz Biotechnology, Dallas, TX, USA) for 30 min at 4°C with rotation. The suspension was then incubated with 7 μl His-Tag Monoclonal antibody (66005-1-Ig, proteintech) for 1 h at 4°C with rotation. About 45 μl protein A/G Plus-Agarose was added and incubated at 4°C with rotation overnight. Beads were then washed five times with 1 ml PBS, and bound proteins were eluted by the addition of 20 μl SDS loading buffer. Boil the samples for 5 min and analyze 10 µl aliquots by western blot.

### IECs isolation

IECs of cecum were isolated as described previously.^62^ In brief, the ceca from the mice were opened longitudinally and washed with PBS to remove intestinal contents. Subsequently, the intestinal segments were incubated in a solution containing 5 mM EDTA, 1 mM DTT, and 5% FBS at 37°C for 15 min. After three times, the medium was collected and centrifuged (1,200 rpm, 5 min) to pellet the cells for subsequent protein extraction.

### Western blot analysis

Protein extracts were separated on 8–15% polyacrylamide gels and then transferred onto polyvinylidene difluoride (PVDF) membranes (MilliporeSigma, Burlington, MA, USA). After blocking nonspecific binding with 5% nonfat dry milk (Sangon Biotech Shanghai Co., Ltd.) membranes were probed with primary antibodies overnight at 4°C and incubated with ppropriate HRP-labeled secondary antibodies at room temperature for 1 h. Membranes were then visualized with an enhanced chemiluminescence luminescence reagent (Meilunbio). The intensities of the bands were determined by ImageJ Launcher broken symmetry software program (NIH, Bethesda, MD, USA). Following antibodies were used: rabbit anti-mouse cleaved caspase-8 (#8592; Cell Signaling Technology), rabbit anti caspase-8 (AC056; Beyotime), Phospho-RSK2 (Ser227) Rabbit mAb (#3556S; Cell Signaling Technology), PDK1 (D37A7) Rabbit mAb (#5662S; Cell Signaling Technology), Caspase-3 (D3R6Y) Rabbit mAb (#14220S; Cell Signaling Technology), Caspase-1 (D7F10) Rabbit mAb (#3866S; Cell Signaling Technology), Anti-MLKL (phospho S345) (ab196436; Abcam), Anti-MLKL (phospho S358) (ab187091; Abcam), Anti-pro Caspase-1 + p10 + p12 (ab179515; Abcam), Anti-GSDMD antibody (ab209845; Abcam), Anti-GSDMD antibody (ab225867; Abcam), Anti-cleaved N-terminal GSDMD (ab215203; Abcam), anti-beta-actin (bs-0061 R; Bioss).

### Quantitative PCR

Total RNA from mouse ceca were isolated using TRIzol reagent (Beyotime Biotechnology, Shanghai, China) according to the manufacturer's protocol and was reverse-transcribed with All-in-one RT MasterMix kit (Applied Biological Materials, Richmond, BC, Canada). qPCR was performed using EvaGreen MasterMix-Low ROX (Applied Biological Materials) in a ViiA7 real-time PCR instrument (Applied Biosystems, Carlsbad, CA, USA). Specific primer sequences are listed in Table 2. All expression levels were normalized to *Gapdh* expression. Values were expressed as fold induction in comparison to untreated control mice.

**Table 2.** Primer sequences for quantitative PCR.

Primers		sequences
*Gapdh*	sense	5’-TGTAGACCATGTAGTTGAGGTCA-3’
	antisense	3’-AGGTCGGTGTGAACGGATTTG-5’
*Il-6*	sense	5’-CTTGGGACTGATGCTGGTGAC-3’
	antisense	3’-GCCATTGCACAACTCTTTTCTC-5’
*Il-1β*	sense	5’-AAAGCCTCGTGCTGTCGGACC-3’
	antisense	3’-CAGGGTGGGTGTGCCGTCTT-5’
*Tnf-α*	sense	5’-GGTGATCGGTCCCCAAAGGGATGA-3’
	antisense	3’-TGGTTTGCTACGACGTGGGCT-5’

### Statistics

Data are presented as mean ± SD. Comparisons among multiple groups were performed with one-way ANOVA. Survival curves were analyzed with log-rank (Mantel–Cox) test. Values of *P* < .05 were considered statistically significant.

## Supplementary Material

Supplemental Material

## Data Availability

The data supporting the findings are openly available at https://doi.org/10.6084/m9.figshare.21813894.v1.

## References

[1] Bakkeren E, Huisman JS, Fattinger SA, Hausmann A, Furter M, Egli A, Slack E, Sellin ME, Bonhoeffer S, Regoes RR, et al. *Salmonella* persisters promote the spread of antibiotic resistance plasmids in the gut. Nature. 2019;573(7773):276–18. doi:10.1038/s41586-019-1521-8. 31485077 PMC6744281

[2] Keestra-Gounder AM, Tsolis RM, Baumler AJ. Now you see me, now you don’t: the interaction of *Salmonella* with innate immune receptors. Nat Rev Microbiol. 2015;13(4):206–216. doi:10.1038/nrmicro3428. 25749454

[3] Raffatellu M, Wilson RP, Chessa D, Andrews-Polymenis H, Tran QT, Lawhon S, Khare S, Adams LG, Baumler AJ, Rauch I, et al. SipA, SopA, SopB, SopD, and SopE2 contribute to *Salmonella enterica* Serotype Typhimurium invasion of epithelial cells. Infect Immun. 2005;73(1):146–154. doi:10.1128/IAI.73.1.146-154.2005. 15618149 PMC538951

[4] Lian H, Jiang K, Tong M, Chen Z, Liu X, Galan JE, Gao X. The *Salmonella* effector protein SopD targets Rab8 to positively and negatively modulate the inflammatory response. Nat Microbiol. 2021;6:658–671. doi:10.1038/s41564-021-00866-3. 33603205 PMC8085087

[5] Fattinger SA, Sellin ME, Hardt WD. *Salmonella* effector driven invasion of the gut epithelium: breaking in and setting the house on fire. Curr Opin Microbiol. 2021;64:9–18. doi:10.1016/j.mib.2021.08.007. 34492596

[6] McGhie EJ. Cooperation between actin-binding proteins of invasive *Salmonella*: sipA potentiates SipC nucleation and bundling of actin. EMBO J. 2001;20(9):2131–2139. doi:10.1093/emboj/20.9.2131. 11331579 PMC125241

[7] Zhou D, Mooseker MS, Galan JE. Role of the *S*. typhimurium Actin-Binding protein *S*ipA in bacterial internalization. Science. 1999;283(5410):2092–2095. doi:10.1126/science.283.5410.2092. 10092234

[8] Demeter A, Jacomin AC, Gul L, Lister A, Lipscombe J, Invernizzi R, Branchu P, Macaulay I, Nezis IP, Kingsley RA, et al. Computational prediction and experimental validation of *Salmonella* Typhimurium SopE-mediated fine-tuning of autophagy in intestinal epithelial cells. Front Cell Infect Microbiol. 2022;12:834895. doi:10.3389/fcimb.2022.834895. 36061866 PMC9428466

[9] Lau N, Haeberle AL, O’Keeffe BJ, Latomanski EA, Celli J, Newton HJ, Knodler LA. SopF, a phosphoinositide binding effector, promotes the stability of the nascent *Salmonella*-containing vacuole. PLoS Pathog. 2019;15(7):e1007959. doi:10.1371/journal.ppat.1007959. 31339948 PMC6682159

[10] Xu Y, Zhou P, Cheng S, Lu Q, Nowak K, Hopp AK, Li L, Shi X, Zhou Z, Gao W, et al. A bacterial effector reveals the V-ATPase-ATG16L1 axis that initiates xenophagy. Cell. 2019;178(3):552–566 e520. doi:10.1016/j.cell.2019.06.007. 31327526

[11] Cheng S, Wang L, Liu Q, Qi L, Yu K, Wang Z, Wu M, Liu Y, Fu J, Hu M, et al. Identification of a novel *Salmonella* type III effector by quantitative secretome profiling. Mol Cell Proteomics. 2017;16(12):2219–2228. doi:10.1074/mcp.RA117.000230. 28887382 PMC5724182

[12] Fattinger SA, Sellin ME, Hardt WD. Epithelial inflammasomes in the defense against *Salmonella* gut infection. Curr Opin Microbiol. 2021;59:86–94. doi:10.1016/j.mib.2020.09.014. 33128958

[13] Fattinger SA, Geiser P, Samperio Ventayol P, Di Martino ML, Furter M, Felmy B, Bakkeren E, Hausmann A, Barthel-Scherrer M, Gul E, et al. Epithelium-autonomous NAIP/NLRC4 prevents TNF-driven inflammatory destruction of the gut epithelial barrier in *Salmonella*-infected mice. Mucosal Immunol. 2021;14(3):615–629. doi:10.1038/s41385-021-00381-y. 33731826 PMC8075861

[14] Hausmann A, Bock D, Geiser P, Berthold DL, Fattinger SA, Furter M, Bouman JA, Barthel-Scherrer M, Lang CM, Bakkeren E, et al. Intestinal epithelial NAIP/NLRC4 restricts systemic dissemination of the adapted pathogen *Salmonella Typhimurium* due to site-specific bacterial PAMP expression. Mucosal Immunol. 2020;13(3):530–544. doi:10.1038/s41385-019-0247-0. 31953493 PMC7181392

[15] Rauch I, Deets KA, Dx J, von Moltke J, Tenthorey JL, Lee AY, Philip NH, Ayres JS, Brodsky IE, Gronert K, et al. NAIP-NLRC4 inflammasomes coordinate intestinal epithelial cell expulsion with eicosanoid and IL-18 release via activation of Caspase-1 and −8. Immunity. 2017;46(4):649–659. doi:10.1016/j.immuni.2017.03.016. 28410991 PMC5476318

[16] Sellin ME, Muller AA, Felmy B, Dolowschiak T, Diard M, Tardivel A, Maslowski KM, Hardt WD. Epithelium-intrinsic NAIP/NLRC4 inflammasome drives infected enterocyte expulsion to restrict *Salmonella* replication in the intestinal mucosa. Cell Host Microbe. 2014;16(2):237–248. doi:10.1016/j.chom.2014.07.001. 25121751

[17] Broz P. Getting Rid of the bad apple: inflammasome-induced extrusion of *Salmonella* -infected enterocytes. Cell Host Microbe. 2014;16(2):153–155. doi:10.1016/j.chom.2014.07.010. 25121744

[18] Samperio Ventayol P, Geiser P, Di Martino ML, Florbrant A, Fattinger SA, Walder N, Sima E, Shao F, Gekara NO, Sundbom M, et al. Bacterial detection by NAIP/NLRC4 elicits prompt contractions of intestinal epithelial cell layers. Proc Natl Acad Sci U S A. 2021;118. doi:10.1073/pnas.2013963118. PMC8072224 33846244

[19] Hefele M, Stolzer I, Ruder B, He GW, Mahapatro M, Wirtz S, Neurath MF, Gunther C. Intestinal epithelial Caspase-8 signaling is essential to prevent necroptosis during *Salmonella* Typhimurium induced enteritis. Mucosal Immunol. 2018;11(4):1191–1202. doi:10.1038/s41385-018-0011-x. 29520026

[20] Vandenabeele P, Galluzzi L, Vanden Berghe T, Kroemer G. Molecular mechanisms of necroptosis: an ordered cellular explosion. Nat Rev Mol Cell Biol. 2010;11(10):700–714. doi:10.1038/nrm2970. 20823910

[21] Orning P, Weng D, Starheim K, Ratner D, Best Z, Lee B, Brooks A, Xia S, Wu H, Kelliher MA, et al. Pathogen blockade of TAK1 triggers caspase-8-dependent cleavage of gasdermin D and cell death. Science. 2018;362(6418):1064–1069. doi:10.1126/science.aau2818. 30361383 PMC6522129

[22] Oberst A, Dillon CP, Weinlich R, McCormick LL, Fitzgerald P, Pop C, Hakem R, Salvesen GS, Green DR. Catalytic activity of the Caspase-8-FLIP(L) complex inhibits RIPK3-dependent necrosis. Nature. 2011;471(7338):363–367. doi:10.1038/nature09852. 21368763 PMC3077893

[23] Zuo H, Chen C, Ma L, Min QX, Shen YH. Caspase-8 knockdown suppresses apoptosis, while induces autophagy and chemo-sensitivity in non-small cell lung cancer cells. Am J Transl Res. 2020;12:6478–6489. 33194045 PMC7653624

[24] Newton K, Wickliffe KE, Dugger DL, Maltzman A, Roose-Girma M, Dohse M, Komuves L, Webster JD, Dixit VM. Cleavage of RIPK1 by Caspase-8 is crucial for limiting apoptosis and necroptosis. Nature. 2019;574(7778):428–431. doi:10.1038/s41586-019-1548-x. 31511692

[25] Young MM, Takahashi Y, Khan O, Park S, Hori T, Yun J, Sharma AK, Amin S, Hu CD, Zhang J, et al. Autophagosomal membrane serves as platform for intracellular death-inducing signaling complex (iDISC)-mediated caspase-8 activation and apoptosis. J Biol Chem. 2012;287(15):12455–12468. doi:10.1074/jbc.M111.309104. 22362782 PMC3320995

[26] Yang ZH, Wu XN, He P, Wang X, Wu J, Ai T, Zhong CQ, Wu X, Cong Y, Zhu R, et al. A Non-canonical PDK1-RSK signal diminishes pro-Caspase-8-mediated necroptosis blockade. Mol Cell. 2020;80(2):296–310 e296. doi:10.1016/j.molcel.2020.09.004. 32979304

[27] Barthel M, Hapfelmeier S, Quintanilla-Martinez L, Kremer M, Rohde M, Hogardt M, Pfeffer K, Russmann H, Hardt WD. Pretreatment of mice with streptomycin provides a *Salmonella enterica* serovar Typhimurium Colitis model that Allows analysis of both pathogen and host. Infect Immun. 2003;71(5):2839–2858. doi:10.1128/iai.71.5.2839-2858.2003. 12704158 PMC153285

[28] Millar AD, Rampton DS, Chander CL, Claxson AW, Blades S, Coumbe A, Panetta J, Morris CJ, Blake DR. Evaluating the antioxidant potential of new treatments for inflammatory bowel disease using a rat model of colitis. Gut. 1996;39(3):407–415. doi:10.1136/gut.39.3.407. 8949646 PMC1383348

[29] Zhang J, Yu Q, Jiang D, Yu K, Yu W, Chi Z, Chen S, Li M, Yang D, Wang Z, et al. Epithelial Gasdermin D shapes the host-microbial interface by driving mucus layer formation. Sci Immunol. 2022;7(68):eabk2092. doi:10.1126/sciimmunol.abk2092. 35119941

[30] Naseer N, Zhang J, Bauer R, Constant DA, Nice TJ, Brodsky IE, Rauch I, Shin S, Raffatellu M. *Salmonella enterica* Serovar Typhimurium induces NAIP/NLRC4- and NLRP3/ASC-independent, Caspase-4-dependent inflammasome activation in human intestinal epithelial cells. Infect Immun. 2022;90(7):e0066321. doi:10.1128/iai.00663-21. 35678562 PMC9302179

[31] Place DE, Lee S, Kanneganti TD. PANoptosis in microbial infection. Curr Opin Microbiol. 2021;59:42–49. doi:10.1016/j.mib.2020.07.012. 32829024 PMC7438227

[32] Tan G, Huang C, Chen J, Chen B, Zhi F. Gasdermin-E-mediated pyroptosis participates in the pathogenesis of Crohn’s disease by promoting intestinal inflammation. Cell Rep. 2021;35(11):109265. doi:10.1016/j.celrep.2021.109265. 34133932

[33] Co JY, Margalef-Catala M, Li X, Mah AT, Kuo CJ, Monack DM, Amieva MR. Controlling epithelial polarity: a human enteroid model for host-pathogen interactions. Cell Rep. 2019;26(9):2509–2520 e2504. doi:10.1016/j.celrep.2019.01.108. 30811997 PMC6391775

[34] Fritsch M, Gunther SD, Schwarzer R, Albert MC, Schorn F, Werthenbach JP, Schiffmann LM, Stair N, Stocks H, Seeger JM, et al. Caspase-8 is the molecular switch for apoptosis, necroptosis and pyroptosis. Nature. 2019;575(7784):683–687. doi:10.1038/s41586-019-1770-6. 31748744

[35] Zhu J, Huang JW, Tseng PH, Yang YT, Fowble J, Shiau CW, Shaw YJ, Kulp SK, Chen CS. From the cyclooxygenase-2 inhibitor celecoxib to a novel class of 3-phosphoinositide-dependent protein kinase-1 inhibitors. Cancer Res. 2004;64(12):4309–4318. doi:10.1158/0008-5472.CAN-03-4063. 15205346

[36] Houles T, Roux PP. Defining the role of the RSK isoforms in cancer. Semin Cancer Biol. 2018;48:53–61. doi:10.1016/j.semcancer.2017.04.016. 28476656

[37] Chen D, Burford WB, Pham G, Zhang L, Alto LT, Ertelt JM, Winter MG, Winter SE, Way SS, Alto NM. Systematic reconstruction of an effector-gene network reveals determinants of *Salmonella* cellular and tissue tropism. Cell Host Microbe. 2021;29(10):1531–1544 e1539. doi:10.1016/j.chom.2021.08.012. 34536347 PMC8516738

[38] Fulde M, van Vorst K, Zhang K, Westermann AJ, Busche T, Huei YC, Welitschanski K, Froh I, Pagelow D, Plendl J, et al. SPI2 T3SS effectors facilitate enterocyte apical to basolateral transmigration of *Salmonella*-containing vacuoles in vivo. Gut Microbes. 2021;13(1):1973836. doi:10.1080/19490976.2021.1973836. 34542008 PMC8475570

[39] Ma S, Liu X, Ma S, Jiang L. SopA inactivation or reduced expression is selected in intracellular *Salmonella* and contributes to systemic *Salmonella* infection. Res Microbiol. 2021;172(2):103795. doi:10.1016/j.resmic.2020.103795. 33347947

[40] Zuo L, Zhou L, Wu C, Wang Y, Li Y, Huang R, Wu S. *Salmonella spvC* gene inhibits pyroptosis and intestinal inflammation to aggravate systemic infection in mice. Front Microbiol. 2020;11:562491. doi:10.3389/fmicb.2020.562491. 33384666 PMC7770238

[41] Knodler LA, Vallance BA, Celli J, Winfree S, Hansen B, Montero M, Steele-Mortimer O. Dissemination of invasive *Salmonella* via bacterial-induced extrusion of mucosal epithelia. Proc Natl Acad Sci U S A. 2010;107(41):17733–17738. doi:10.1073/pnas.1006098107. 20876119 PMC2955089

[42] Geiser P, Di Martino ML, Samperio Ventayol P, Eriksson J, Sima E, Al-Saffar AK, Ahl D, Phillipson M, Webb DL, Sundbom M, et al. *Salmonella enterica* Serovar Typhimurium exploits cycling through epithelial cells to colonize human and murine enteroids. mBio. 2021;12. doi:10.1128/mBio.02684-20. PMC7844539 33436434

[43] Wotzka SY, Nguyen BD, Hardt WD. *Salmonella* Typhimurium diarrhea reveals basic principles of enteropathogen infection and disease-promoted DNA exchange. Cell Host Microbe. 2017;21(4):443–454. doi:10.1016/j.chom.2017.03.009. 28407482

[44] Galan JE. *Salmonella* Typhimurium and inflammation: a pathogen-centric affair. Nat Rev Microbiol. 2021;19(11):716–725. doi:10.1038/s41579-021-00561-4. 34012042 PMC9350856

[45] Lee BL, Mirrashidi KM, Stowe IB, Kummerfeld SK, Watanabe C, Haley B, Cuellar TL, Reichelt M, Kayagaki N. ASC- and caspase-8-dependent apoptotic pathway diverges from the NLRC4 inflammasome in macrophages. Sci Rep. 2018;8(1):3788. doi:10.1038/s41598-018-21998-3. 29491424 PMC5830643

[46] Van Opdenbosch N, Van Gorp H, Verdonckt M, Saavedra PHV, de Vasconcelos NM, Goncalves A, Vande Walle L, Demon D, Matusiak M, Van Hauwermeiren F, et al. Caspase-1 engagement and TLR-Induced c-FLIP expression suppress ASC/Caspase-8-dependent apoptosis by inflammasome sensors NLRP1b and NLRC4. Cell Rep. 2017;21(12):3427–3444. doi:10.1016/j.celrep.2017.11.088. 29262324 PMC5746600

[47] Dong K, Zhu Y, Deng Q, Sun L, Yang S, Huang K, Cao Y, Li Y, Wu S, Huang R. *Salmonella* pSLT-encoded effector SpvB promotes RIPK3-dependent necroptosis in intestinal epithelial cells. Cell Death Discov. 2022;8(1):44. doi:10.1038/s41420-022-00841-9. 35110556 PMC8810775

[48] Lin HH, Chen HL, Weng CC, Janapatla RP, Chen CL, Chiu CH. Activation of apoptosis by *Salmonella* pathogenicity island-1 effectors through both intrinsic and extrinsic pathways in *Salmonella*-infected macrophages. J Microbiol Immunol Infect. 2021;54(4):616–626. doi:10.1016/j.jmii.2020.02.008. 32127288

[49] Newson JPM, Scott NE, Yeuk Wah Chung I, Wong Fok Lung T, Giogha C, Gan J, Wang N, Strugnell RA, Brown NF, Cygler M, et al. *Salmonella* effectors SseK1 and SseK3 target death domain proteins in the TNF and TRAIL signaling pathways. Mol Cell Proteomics. 2019;18(6):1138–1156. doi:10.1074/mcp.RA118.001093. 30902834 PMC6553940

[50] Gunster RA, Matthews SA, Holden DW, Thurston TLM. SseK1 and SseK3 type III secretion system effectors inhibit NF-κB signaling and necroptotic cell death in *Salmonella*-infected macrophages. Infect Immun. 2017:85. doi:10.1128/IAI.00010-17. PMC5328493 28069818

[51] Han JH, Park J, Kang TB, Lee KH. Regulation of Caspase-8 activity at the crossroads of pro-inflammation and anti-inflammation. Int J Mol Sci. 2021;23(1):22. doi:10.3390/ijms22073318. 33805003 PMC8036737

[52] Hu GQ, Yang YJ, Qin XX, Qi S, Zhang J, Yu SX, Du CT, Chen W. *Salmonella* outer protein B suppresses colitis development via protecting cell from necroptosis. Front Cell Infect Microbiol. 2019;9:87. doi:10.3389/fcimb.2019.00087. 31024858 PMC6465518

[53] Garcia-Gil A, Galan-Enriquez CS, Perez-Lopez A, Nava P, Alpuche-Aranda C, Ortiz-Navarrete V. SopB activates the Akt-YAP pathway to promote *Salmonella* survival within B cells. Virulence. 2018;9(1):1390–1402. doi:10.1080/21505594.2018.1509664. 30103648 PMC6177241

[54] Hu GQ, Song PX, Chen W, Qi S, Yu SX, Du CT, Deng XM, Ouyang HS, Yang YJ. Critical role for *Salmonella* effector SopB in regulating inflammasome activation. Mol Immunol. 2017;90:280–286. doi:10.1016/j.molimm.2017.07.011. 28846926

[55] Levina A, Fleming KD, Burke JE, Leonard TA. Activation of the essential kinase PDK1 by phosphoinositide-driven trans-autophosphorylation. Nat Commun. 2022;13(1):1874. doi:10.1038/s41467-022-29368-4. 35387990 PMC8986801

[56] Alfonso M, David K, Daan MF, Dario RA. PDK1, the master regulator of AGC kinase signal transduction. Semin Cell Dev Biol. 2004;15(2):161–170. doi:10.1016/j.semcdb.2003.12.022. 15209375

[57] Qu Y, Misaghi S, Izrael-Tomasevic A, Newton K, Gilmour LL, Lamkanfi M, Louie S, Kayagaki N, Liu J, Komuves L, et al. Phosphorylation of NLRC4 is critical for inflammasome activation. Nature. 2012;490(7421):539–542. doi:10.1038/nature11429. 22885697

[58] Mauvezin C, Neufeld TP. Bafilomycin A1 disrupts autophagic flux by inhibiting both V-ATPase-dependent acidification and Ca-P60A/SERCA-dependent autophagosome-lysosome fusion. Autophagy. 2015;11(8):1437–1438. doi:10.1080/15548627.2015.1066957. 26156798 PMC4590655

[59] Wu S, Shen Y, Zhang S, Xiao Y, Shi S. *Salmonella* interacts with autophagy to offense or defense. Front Microbiol. 2020;11:721. doi:10.3389/fmicb.2020.00721. 32390979 PMC7188831

[60] Mandal R, Barron JC, Kostova I, Becker S, Strebhardt K. Caspase-8: the double-edged sword. Biochim Biophys Acta Rev Cancer. 2020;1873(2):188357. doi:10.1016/j.bbcan.2020.188357. 32147543

[61] Choi CY, Vo MT, Nicholas J, Choi YB. Autophagy-competent mitochondrial translation elongation factor TUFM inhibits Caspase-8-mediated apoptosis. Cell Death Differ. 2022;29(2):451–464. doi:10.1038/s41418-021-00868-y. 34511600 PMC8817016

[62] Nenci A, Becker C, Wullaert A, Gareus R, van Loo G, Danese S, Huth M, Nikolaev A, Neufert C, Madison B, et al. Epithelial NEMO links innate immunity to chronic intestinal inflammation. Nature. 2007;446(7135):557–561. doi:10.1038/nature05698. 17361131

