# Supplementary figures and images for: Salmonella effector SopF regulates PANoptosis of intestinal epithelial cells to aggravate systemic infection
Source: Gut Microbes. 2023 Feb 20;15(1):2180315. doi: 10.1080/19490976.2023.2180315 (PMC9980482; doi:10.1080/19490976.2023.2180315)

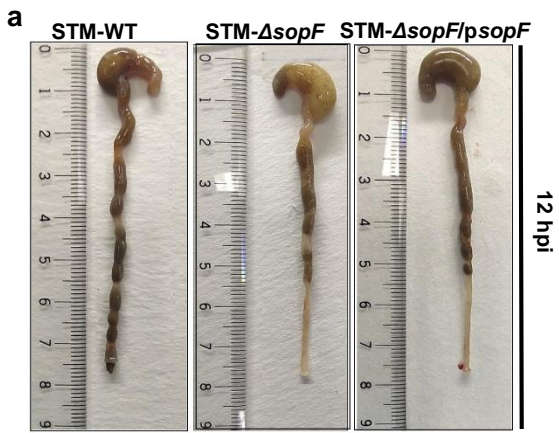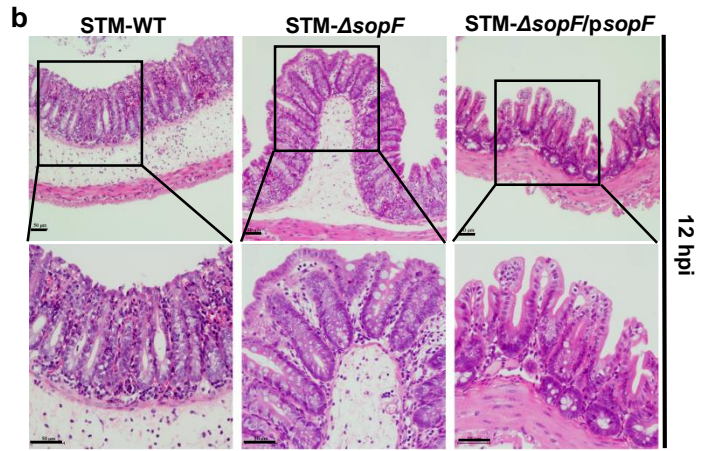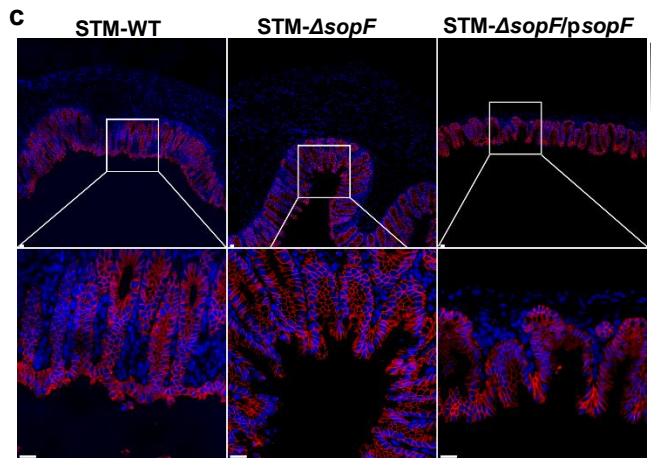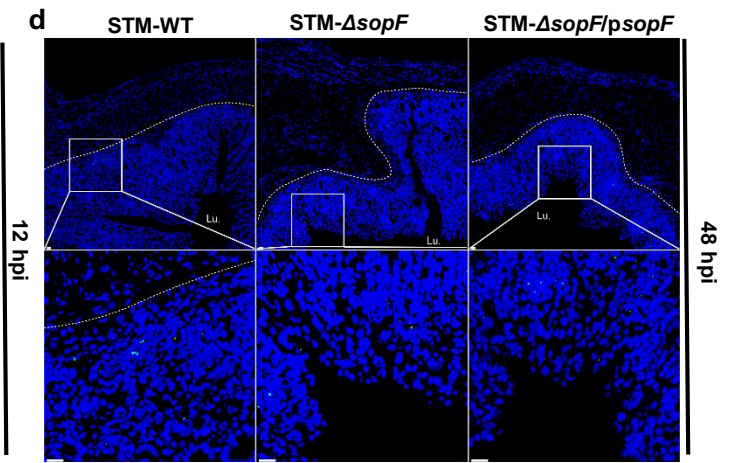

Supplement: Supplemental Material [file KGMI_A_2180315_SM7405.zip › FigS1.pdf]

# PANoptosis in NCM460

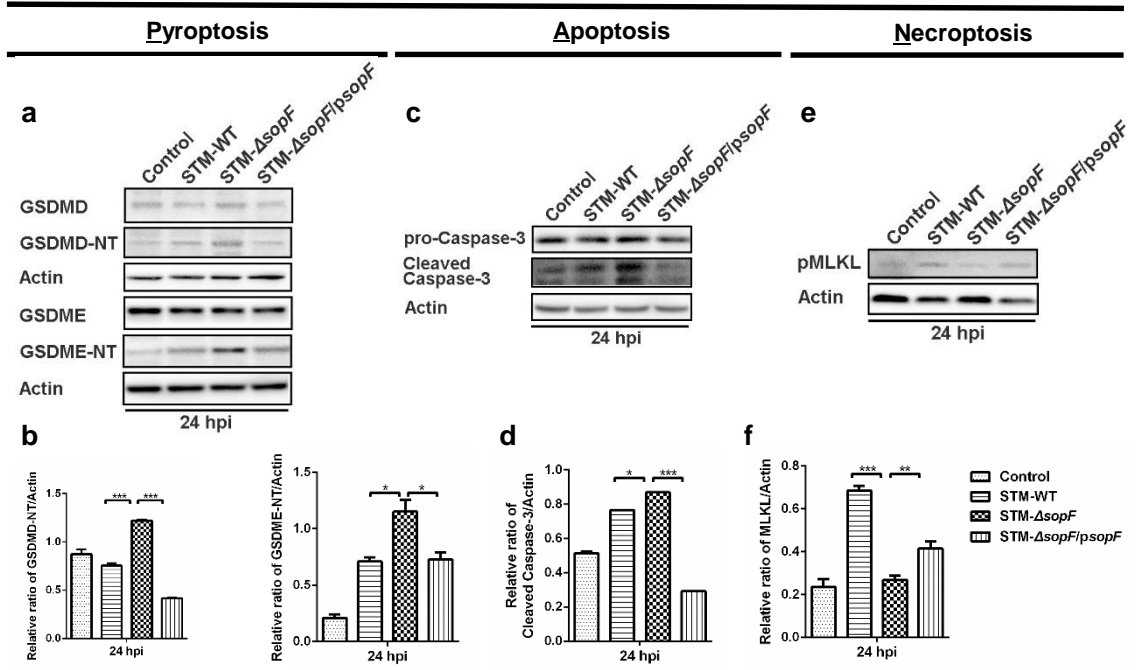

Supplement: Supplemental Material [file KGMI_A_2180315_SM7405.zip › FigS2.pdf]

**a**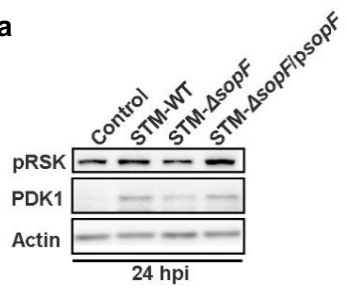**b**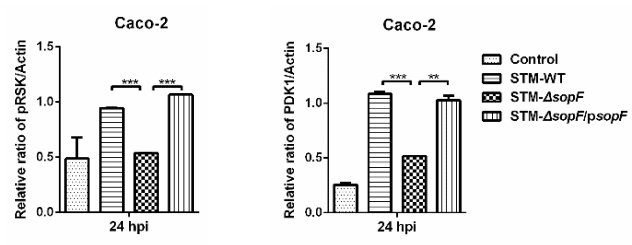**c**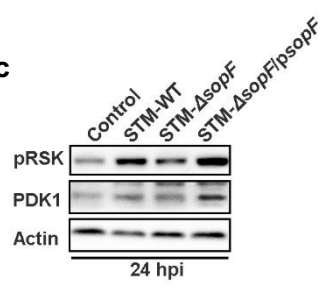**d**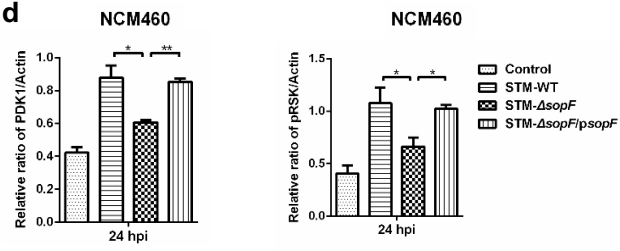

Supplement: Supplemental Material [file KGMI_A_2180315_SM7405.zip › FigS3.pdf]

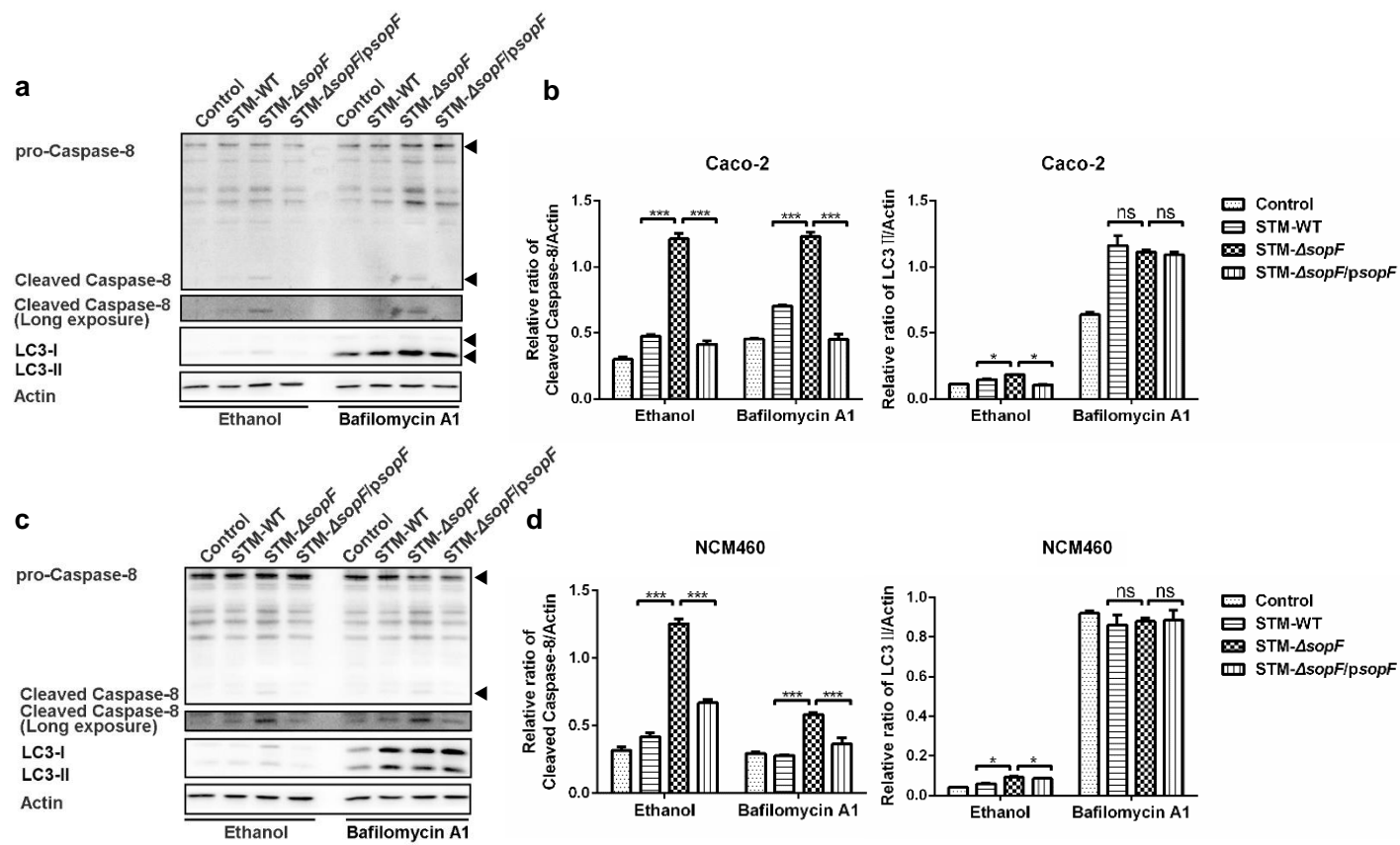

Supplement: Supplemental Material [file KGMI_A_2180315_SM7405.zip › FigS4.pdf]
